# Manipulation of P2X Receptor Activities by Light Stimulation

**DOI:** 10.1155/2016/7852168

**Published:** 2016-01-17

**Authors:** Sang Seong Kim

**Affiliations:** Department of Pharmacy, Hanyang University, ERICA Campus, 55 Hanyangdaehak-ro, Sangnok-gu, Ansan, Gyeonggi-do 426-791, Republic of Korea

## Abstract

P2X receptors are involved in amplification of inflammatory responses in peripheral nociceptive fibers and in mediating pain-related signals to the CNS. Control of P2X activation has significant importance in managing unwanted hypersensitive neuron responses. To overcome the limitations of chemical ligand treatment, optical stimulation methods of optogenetics and photoswitching achieve efficient control of P2X activation while allowing specificity at the target site and convenient stimulation by light illumination. There are many potential applications for photosensitive elements, such as improved uncaging methods, photoisomerizable ligands, photoswitches, and gold nanoparticles. Each technique has both advantages and downsides, and techniques are selected according to the purpose of the application. Technical advances not only provide novel approaches to manage inflammation or pain mediated by P2X receptors but also suggest a similar approach for controlling other ion channels.

## 1. Introduction

P2X receptors belong to the ATP-gated cation channel family, which has seven members [1–8]. The receptor structure consists of trimeric homo- or heteromers with two transmembrane domains (TM1 and TM2) [2–4]. P2X receptors have various roles in neuropathic pain [5, 6], synaptic transmission [2], cancer [7], neurodegenerative disorders [8], and inflammation [9–12]. Release of ATP during inflammation is characteristic at the site of injury. Activated inflammatory cells release a plethora of ATP extracellularly in an uncontrolled fashion from intracellular storage [13, 14]. In some cases, controlled release of ATP is observed through transmembrane proteins connexin or pannexin [13, 15]. ATP also functions as a neurotransmitter in primary afferent neurons by relaying pain information to the CNS [16]. Considering the distribution of P2X, its involvement in nociceptive sensation cannot be ruled out. P2X1 to P2X6 are expressed in sensory ganglia, especially in dorsal root ganglia (DRG) [17]. The dense expression of P2X3 in small diameter DRG and its coexpression with TRPV1 implicate P2X3 as a designated pain sensor [18, 19]. Thus, activation of P2X in inflammatory or noxious conditions provokes widespread responses in neuropathic pain. Nerve damage induces upregulation of P2X receptors, leading to hypersensitivity [20].

Considering the fundamental contribution of P2X receptors in pain and inflammation, receptor modulation has therapeutic significance. Traditionally, chemical compounds are applied to local tissues or systemic circulation to control ion channel activity. Oral intake, stable structure in ordinary conditions, and high efficacy at target sites are advantages of using chemical drugs. However, side effects resulting from systemic distribution and harmful metabolite production require improved approaches. Due to advances in the optogenetic field in the last decade, novel techniques have been introduced to control ion channel activity. The use of photoswitches or genetically modified receptors has increased specificity in controlling target channel activities with optimal light stimulation. In addition to optogenetics, the incorporation of novel materials into cells has enabled optimal control of intact receptors without genetic modifications. These techniques encompass important target channels or receptors involved in neuronal firing, inflammation, or incurable diseases. P2X receptors are targets of interest due to their important role in inflammation and nociceptive sensation. This review investigates past trials on control of P2X receptors by light stimulation and explores cutting edge techniques with potential for therapeutic application.

## 2. Activation of P2X Receptors by Releasing ATP from Photosensitive Caging Compounds

In conventional neuronal stimulation, an electric stimulus is delivered through an electric probe to activate the group of neurons surrounding the probe. This direct electrical activation is easily applicable with a potential caveat of nonspecific activation of neuronal circuits. P2X receptors have been favored for optical stimulation due to their simple channel architecture, large extracellular ligand binding domain, and relatively rare presence in the CNS [21–24]. The initial application was with an uncaging method that tethered ATP to caging compounds as a protecting group. The first generation was 2-nitrobenzyl (NB) esters of ATP that were susceptible to photolysis by UV light [25, 26]. The side effect of NB photolysis, however, produced reactive by-product compounds that interfered with reliable interpretation of the response. Improved second-generation caging groups, such as 4,5-dimethoxy-2-nitrobenzyl (DMNB) and *α*-carboxy-2-nitrobenzyl (CNB), overcame these limitations with fewer by-products and stable kinetics [27]. Indeed, robust neuronal spikes were generated in P2X2 expressing neurons using a 1 s UV light pulse (355 nm) in the presence of DMNPE-ATP [28]. P2X2 activation kinetics fell into the millisecond range in a reproducible manner, suggesting applications in spatially restricted sites to invoke the intended neuronal activation. This scenario was realized in phototrigger experiments affecting the locomotor behavior of flies. Lima and Miesenböck expressed P2X2 in a small group of neurons of the giant fiber (GF) system through DMNPE-ATP injection in the CNS [29]. Brief UV illumination for 150–250 ms provoked characteristic escape movements mediated through giant fibers without desensitization in repeated photostimulation of 2.5 s intervals. The induction of dopaminergic signaling by photostimulation of P2X2 expressing dopaminergic neurons increased locomotor activities affecting the walking pattern of flies. Likewise, remote control of P2X activation by photostimulation not only changed the cellular response but also caused behavioral changes.

## 3. Control of Neuronal Activation by Photoisomerizable Molecules Transporting through P2X Receptor Pores

The optochemical genetic approach has downsides in therapeutic applications due to exogenous expression of effective receptors and systemic injection of caging compounds, which generates nonselective cellular responses. To circumvent these problems, photoisomerizable molecules were introduced through certain receptor pores. They then underwent conformational changes induced by light stimulation. This idea was originally developed to silence nociceptive neurons by delivering membrane-impermeable local anesthetic lidocaine, QX-314, intracellularly with the help of TRPV1 pore opening [30]. The attempt was successful in allowing QX-314 influx by TRPV1 activation, but structural irreversibility of the compound lasted for several hours, preventing therapeutic usage [31].

To overcome this limitation, Mourot et al. developed a novel photoswitch, quaternary ammonium-azobenzene-quaternary ammonium (QAQ). QAQ has versatile isomerizable characteristics and converts from the elongated* trans*  form to bent* cis *form with 380 nm light [32]. Once introduced intracellularly, the* trans* form of QAQ blocks various voltage-gated Na^+^, Ca^2+^, and K^+^ channels with the few exceptions of inward-rectifier (Kir), hyperpolarization-activated cyclic nucleotide-gated (HCN) channels,* N*-methyl-D-aspartic acid (NMDA), and non-NMDA receptors. Light stimulation of 380 nm on QAQ embedded neurons recovered the silenced nociceptive neurons, restoring their firing rate and action potential amplitude. Light of 500 nm generated the opposite effect. The transition with light of specific wavelengths was evoked in an on and off fashion without altering basic neuronal properties such as resting membrane potential or firing threshold. QAQ can enter cells through large ionic pores, which limits the number of mediator channels or receptors. TRPV1 expressing cells showed sufficient QAQ loading in the presence of capsaicin [32]. P2X7 also proved its eligibility for photosensitization with ATP treatment [32, 33]. In heterologous expression of HEK 293 cells, P2X7 demonstrated photosensitization of Shaker K^+^ channels by QAQ application in the presence of ATP. Endogenous voltage-gated K^+^ channel activity was also prone to photocontrol through exogenously expressed P2X7 receptors in cultured rat hippocampal neurons.

This novel photoswitching technology is applicable in pain and inflammation control due to the immense role of TRPV1 and P2X7 in pathological processes. Because QAQ has selective entry without affecting nonnociceptive neurons, it provides an effective peripheral analgesic method. QAQ can enter any nociceptor location from the cell soma to the long axonal process and nerve endings through pores of mediating channels, which is impossible with conventional electrophysiology or drugs.

## 4. Optogating of P2X Receptors through Introduction of Photoswitches into the Subunits 

With recent advances in optogenetic technology, remote control of neuronal activity enables selective activation of certain neuronal circuits, leading to local or systemic changes on demand. Among various applications, pharmacology takes advantage of photoswitchable ligands, which are tethered to genetically modified receptor sites. Photoisomerizable chemical ligands can convert geographical structures into more favorable forms by light stimulation with a specific wavelength, enabling binding to active sites of receptors. This method improved conventional chemical stimulation by increasing the specificity of target locations and instant stimulation.

Efforts have been made to enable optogating of P2X receptors. An azobenzene ammonium derivative was synthesized to create a maleimide ethylene azobenzene trimethyl ammonium derivative (MEA-TMA). This was photoisomerized from the long* trans*-isomer to the* cis*-isomer by applying a 365 nm light, a change that was reversed with 525 nm light [34]. MEA-TMA was affixed to a modified P2X receptor, P2X2-3T, in which outer transmembrane residues were substituted to cysteine [35]. Indeed, 365 nm light precisely opened the channel within a short temporal range, reaching maximal current in almost 1 second. The optogating of P2X2 consisted of two different mechanisms, an ATP dependent pore-blocking pathway and direct binding to the receptor without ATP. With ATP independent opening, a pore dilation effect was still observed with* N*-methyl-D-glucamine (NMDG) treatment [2, 36, 37].

A similar attempt using azobenzene isomerization was made for optical control of p2X2 and P2X3 receptors. In this experiment,* bis*(maleimido)azobenzene (BMA) was synthesized to bridge receptor subunits of TM2 through covalent linkage to cysteine residues [38]. BMA underwent conformational changes between the* cis* and* trans *forms at 360 and 440 nm illumination, respectively. P2X2 receptor P329C mutation with BMA treatment allowed an inward current with 440 nm in the absence of ATP. Cysteine substitution of P320C at an equivalent site of P2X3 also generated light-activated current, as with P2X2 activation. Homomeric expression of each channel and P2X2/3 heteromeric expression showed rapid photocontrol with opening at 440 nm and closing at 360 nm in HEK293 cells and PC12 cells. Photoisomerization showed similar receptor kinetics as ATP activation. This result suggests that gating rearrangement is the limiting factor of P2X activation, not agonist-binding steps.

## 5. Neuronal Activation Using Gold Particles to Target Intact P2X Receptors

Various methods have been suggested to achieve specific optical stimulation of neurons, as described above. P2X receptor activation photoswitch or photoisomerization offers custom designed activation tools. However, genetic modification of receptors is inevitable. Actual application in nongenetically modified animals, including humans, is hindered. To overcome this limitation, direct optical stimulation of intact neurons is proposed by applying infrared (IR) wavelength to neuronal membranes [39, 40]. IR wavelength delivers energy in the form of heat to cellular membranes, leading to upregulation of membrane capacitance. This elevated membrane capacitance results in depolarization and action potential generation.

To effectively deliver heat to cell membranes, gold nanorods (AuNRs) or spherical gold nanoparticles (AuNPs) are treated in cell culture media to absorb IR energy and convert it to heat. In this way, cultured neurons can evoke cellular activation by IR or near-infrared (NIR) excitation [41–43]. Bezanilla et al. elaborated upon this method by using channel specific antibodies and second antibodies conjugated with AuNPs to specifically activate neurons expressing target channels [44]. They initially tested dorsal root ganglion (DRG) neurons with Ts1-conjugated AuNPs. Light of 532 nm was sufficient to generate action potentials in DRG neurons, even with repetitive washout and multiple photostimulations. The next trial targeted endogenously expressed channels such as TRPV1 and P2X3. Overnight incubation of DRG neurons with each antibody following AuNP-secondary antibody treatment demonstrated the photosensitivity of DRG neurons to green light stimulation (532 nm). The amplitude of action potentials generated by light stimulation was almost equal to that of electrical stimulation. Long washout time of more than 20 min still preserved responsiveness to light, proving tight binding of primary antibodies. This method encourages clinical applications to control nociceptive neuronal activation in pain or inflammation. Unlike any other technique, this method does not require injection of a virus containing modified receptor DNA at the site of interest. Instead, simple treatment with AuNP-conjugated antibody and NIR illumination is sufficient to activate target neurons. Since the activation mechanism does not depend on temperature itself but rather on the rate of temperature change, less intense light stimulation can be applied to avoid tissue damage.

## 6. Conclusion

Growth in optogenetics has generated abundant opsin containing channels with ample wavelength ranges of light stimulation to activate or deactivate neurons. Channelrhodopsins and halorhodopsins are the most representative light-gated ion channels, and each intracellularly introduces cations and anions. Genetic expression of these photosensitive channels using viral vectors is helpful for controlling neuronal activities. However, introduction of foreign genes can cause derangements in proper protein expression. Therefore, receptors already present in nerves are safer targets. P2X receptors are choice candidates for light stimulation because they are functionally expressed in the periphery and CNS to govern inflammatory and nociceptive pathways. For a long time, uncaging of ATP by UV light has been the primary method used to activate P2X receptors. Improved caging compounds are now used to reduce by-products. The isomerizable property of azobenzene introduced a new way to construct photoswitches that can reversibly change conformations. Photoswitches can be incorporated into receptor subunits or used as light sensitive ligands. Controlling both receptors and ligands by light stimulation enables many options for combinatorial manipulation of neuronal activation. Even intact receptors can receive light stimulation. The novel concept of applying gold particles to convert light energy into heat proved the possibility of activating neurons by increasing membrane conductance. Using specific antibodies for pain receptors such as P2X, gold nanoparticles target intact receptors to activate nerves.

Although there are diverse benefits with light stimulation techniques, every method inevitably introduces limitations also. Our goal is to improve current methods to increase specificity while developing safer applications in humans. The fields of optics and material science suggest innovative technical advances for new ideas.
